# Reverse immunodynamics: a new method for identifying targets of protective immunity

**DOI:** 10.1038/s41598-018-37288-x

**Published:** 2019-02-15

**Authors:** Katrina J. Spensley, Paul S. Wikramaratna, Bridget S. Penman, Andrew Walker, Adrian L. Smith, Oliver G. Pybus, Létitia Jean, Sunetra Gupta, José Lourenço

**Affiliations:** 1 Imperial College London, London, W2 1PG UK; 2 Institute of Evolutionary Biology, University of Edinburgh, Edinburgh, EH9 3JT UK; 3 Department of Zoology, University of Oxford, Oxford, OX1 3PS UK; 4 The Sir William Dunn School of Pathology, University of Oxford, Oxford, OX1 3RE UK; 5 School of Life Sciences, University of Warwick, Coventry, CV4 7AL UK

## Abstract

Despite a dramatic increase in our ability to catalogue variation among pathogen genomes, we have made far fewer advances in using this information to identify targets of protective immunity. Epidemiological models predict that strong immune selection can cause antigenic variants to structure into genetically discordant sets of antigenic types (e.g. serotypes). A corollary of this theory is that targets of immunity may be identified by searching for non-overlapping associations of amino acids among co-circulating antigenic variants. We propose a novel population genetics methodology that combines such predictions with phylogenetic analyses to identify genetic loci (epitopes) under strong immune selection. We apply this concept to the AMA-1 protein of the malaria parasite *Plasmodium falciparum* and find evidence of epitopes among certain regions of low variability which could render them ideal vaccine candidates. The proposed method can be applied to a myriad of multi-strain pathogens for which vast amounts of genetic data has been collected in recent years.

## Introduction

Among pathogens for which protective immune responses are directed against conserved targets (epitopes), a single infection ensures lifelong immunity, and the goal of vaccination is to elicit natural-like immunity against such conserved targets. Most infectious diseases against which we have effective vaccines fall into this category (e.g. measles). By contrast, the primary targets of protective immunity in several important pathogens such as *Plasmodium falciparum*, the human immunodeficiency virus (HIV) or the influenza A virus, are highly diverse and present a challenge to the development of vaccines.

One solution to this problem that is currently being explored by several research groups, is the artificial boosting of immunity towards conserved regions that are not sufficiently naturally immunogenic to prevent further infection upon single exposure^1,2^. Another solution is to target naturally immunogenic epitopes of very high variability (e.g. influenza A), with the drawback that vaccines may need recurrent updating. As opposed to targeting conserved epitopes of low immunogenicity or highly variable epitopes of high immunogenicity, an alternative and less researched strategy is to target epitopes of limited variability which are under immune selection. This strategy may allow to develop multi-allelic vaccines in which immunogenic epitopes that confer protection against a sub-population of strains are combined to provide broad coverage (e.g. Thompson *et al*.^3^).

In this study, we attempt to identify low variability epitopes under strong immune selection by using a novel approach based on the predictions of a multi-locus mathematical framework of pathogen evolution commonly referred to as *strain theory*^4–6^. The central premise we focus on, is that regions (or epitopes) under strong immune pressure are selected to self-organise into genetically discordant sets of antigenic types (e.g. dengue virus, meningococcal or pneumococcal serotypes).

This prediction is so far supported by empirical observations within a variety of host-pathogen systems^7–13^. In our proposed approach, we invert this prediction to search for signatures of immune selection by identifying genetic regions which exhibit a high degree of non-overlap, but critically excluding those that may have arisen as a consequence of shared ancestry or due to structural interactions. We term this novel approach the *reverse immunodynamic* method and present proof of concept results from a vast number of genetic sequences of the apical membrane antigen 1 protein (AMA-1).

AMA-1 is a trans-membrane protein common to all *Plasmodium* species with a putative role in invasion at erythrocytic and pre-erythrocytic stages of the parasite life-cycle. Antibodies to AMA-1 appear to inhibit erythrocyte invasion and are thus thought to play a role in clinical protection against both *P*. *falciparum*^14–20^, *P*. *knowlesi*^21^ and *P*. *vivax*^22^. Cellular responses to AMA-1 may provide immunity against both erythrocytic^23^ and pre-erythrocytic forms of *P*. *falciparum*^24^. The potential of AMA-1 as a component of a multi-stage malaria vaccine is compromised by the polymorphism of many of the targets of natural immunity^25,26^ as well as by difficulties in inducing lasting immune responses through vaccination^27–30^. The extracellular region of AMA-1 can be structurally resolved into three domains: DI, DII and DIII. The highest level of polymorphism is seen among residues in DI and DIII surrounding a conserved hydrophobic trough^31–36^ which plays a vital role in the invasion process^37–39^. Antibodies against this region have been shown to block invasion in a strain-specific manner; however, it is also clear that these antibodies constitute only a small proportion of the total inhibitory humoral response^26,40,41^. Identifying low variability regions of AMA-1 that are under strong immune selection would afford the possibility of focusing vaccine-induced immune responses on these regions and ensure full coverage of variability at these critical epitopes.

Previous studies on AMA-1 genetic data have demonstrated that selection pressure as measured by standard population genetics measures is strongest within DI and DIII^34–36^, and that while these are also highly variable, they contain a number of less variable residues^35,36^. Using our proposed *reverse immunodynamics* method we expand on these studies by detecting strong signatures of non-overlap among a small number of dimorphic residues in DIII, thus identifying epitopes within that wide region which are targets of protective immunity.

## Results

We implemented the reverse immunodynamic method by performing pairwise comparisons of all sites among 1198 unique PfAMA-1 sequences obtained from GenBank, to determine whether any of these pairs exhibited non-overlapping associations between amino acid residues (see Methods section for details). We used an Information Theory statistic known as Mutual Information (MI), scaled to its maximum value (scaled MI), to measure the degree of non-overlap in amino acid combinations between pairs of sites of PfAMA-1 sequences (see Supplementary Text 1 for mathematical formulations and theoretical background). The main results are focused on dimorphic sites as we aim at the discovery of low variability epitopes, but output of our approach for all polymorphic pairs of sites is made available in Table S1.

### A limited number of site-pairs exhibit high non-overlap

**Figure 1:**
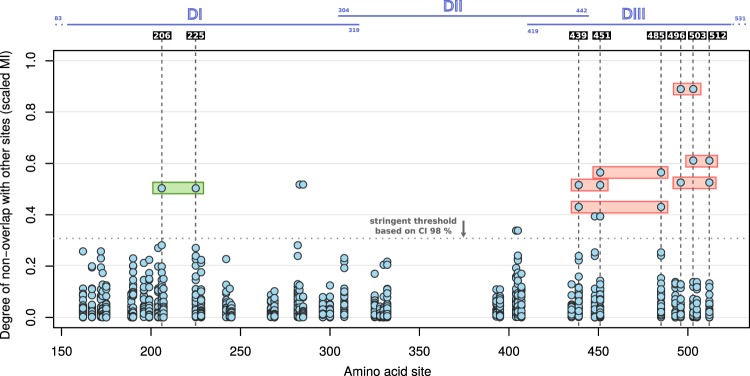
Degree of non-overlap between amino acid residues at pairs of dimorphic sites. Each site on the x-axis (positions 149–534) that is dimorphic is compared with all other dimorphic sites. Those with high scores above a stringent scaled MI threshold are shown by bars spanning both sites (red and green). Scaled MI threshold is based on the top 1% of observed scaled MI values (CI 98% of the observed distribution, upper limit of 0.312, horizontal dotted grey line). Red bars mark pairs of sites of interest, and the green bar marks the pair that is not of interest once phylogenetic relationships are taken into account.

Most pairs of sites showed low non-overlap, with only 10 pairs exhibiting a scaled MI in excess of 0.312 (Table 1), a threshold based on the top 1% of the distribution of all observed scaled MI values (upper limit of a stringent 98% confidence interval, CI) (Table S2). Of these pairs, 404/405, 448/451 and 283/285 were eliminated on grounds of sequence proximity based on the lower limit (d = 3) 95% CI of all observed distances between dimorphic pairs. Figure 1A highlights the remaining 7 pairs among all the background scaled MI measures.

**Table 1:** Counts of amino acid associations between pairs of sites with high non-overlap (scaled MI > 0.312).

Association	Amino Acid Combinations				Scaled MI
496 vs 503	**IN**	**IR**	**MN**	**MR**	0.89
	602	3	14	561	
503 vs 512	**NK**	**NR**	**RK**	**RR**	0.61
	565	52	40	524	
451 vs 485	**KI**	**KK**	**MI**	**MK**	0.57
	604	103	15	476	
496 vs 512	**IK**	**IR**	**MK**	**MR**	0.53
	552	68	54	523	
283 vs 285	**LE**	**LQ**	**SE**	**SQ**	0.52
	242	71	1	883	
439 vs 451	**HK**	**HM**	**NK**	**NM**	0.5
	660	72	46	390	
206 vs 225	**EI**	**EN**	**KI**	**KN**	0.5
	96	806	290	6	
439 vs 485	**HI**	**HK**	**NI**	**NK**	0.41
	588	144	30	406	
448 vs 451	**DK**	**DM**	**NK**	**NM**	0.39
	705	196	2	295	
404 vs 405	**RE**	**RK**	**TE**	**TK**	0.34
	108	635	367	84

### For most pairs, high non-overlap is unlikely to have arisen by neutral evolutionary processes

**Figure 2:**
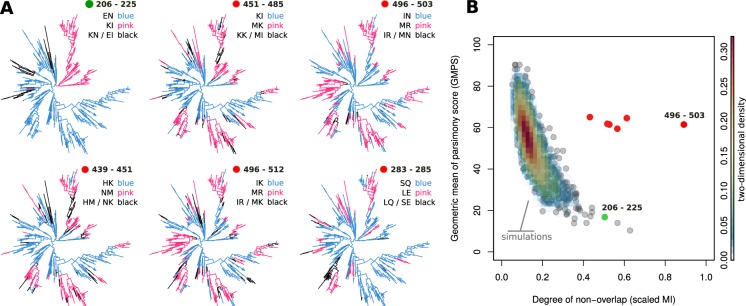
Empirical phylogenetic allelic structure, simulated parsimony score and scaled MI. (**A**) Example, ML phylogenetic trees of AMA-1 with branches coloured on the basis of observed combination of amino acid variants found at tips for different pairs of sites. Pink and cyan are used for the two most common amino acid variants (which are discordant for these sites, see Table 1) and black for the least common. **(B)** Empirical pairs of sites described in Fig. 1 and Table 1 are shown in relation to a null distribution of site-pairs from 1000 simulations that scored most highly (rank 1) on S-score (the product of scaled MI measuring non-overlap, and geometric mean of parsimony score indicating the minimum number of genetic changes required to explain the observed ancestral relationships, GMPS). Color key (scale) on the right is the two-dimensional density of GMPS and scaled MI of the simulated site-pairs. **(A**,**B)** Pairs of sites identified in red and green in the two panels match the pairs in Fig. 1.

To assess whether high non-overlap between sites could have arisen by neutral evolutionary processes (e.g. ancestry), we started by estimating a maximum likelihood (ML) phylogenetic tree from the sequences (see Methods for details). A major difference became evident upon inspection of the phylogenetic allelic structure of site-pair 206/225 compared to the other pairs of interest (Fig. 2A). As is visually shown, the dominant non-overlapping pairs of amino acids at 206/225 are highly segregated and present an ancient dimorphic relationship, which has been observed in other *P*. *falciparum* genes such as DBLMSP/DBLMSP2^33^, MSP1^42^, or MSP2^43^. The other pairs of sites exhibited a more interdigitated pattern, indicating multiple independent changes.

To try to quantify these differences, we calculated the parsimony score (PS) for each site (see Supplementary Text 1 for details), which is the minimum number of genetic changes required to explain the observed ancestral relationships (in this case, back and forth between the two possible amino acids at each site). We then calculated the product of scaled MI and the geometric mean of PS (GMPS) for each site, which we termed the S-score. Importantly, this new measure was found to preserve the same order as the scaled MI scores of the 7 pairs of interest (Table 1), except in the case of the 206/225 (values in Table S2). This meant that six out of seven pairs of sites had both intermediate-to-high scaled MI and GMPS values.

To obtain a null distribution of the S-score for hypothesis testing, we simulated 1000 sets of sequences which were (a) consistent with the ancestry of the empirical phylogenetic tree, and (b) had the same PS score at each dimorphic site as the empirical data (see Methods). Given that six out of seven pairs of sites of interest had the highest empirical S-scores, we selected, from each of the 1000 simulations, the highest S-scored pair of sites (termed rank 1 pairs, values in Table S4). The two-dimensional distribution of rank 1 pairs of sites (Fig. 2B, grey points) showed that neutral evolution can generate sites with high PS or high scaled MI values, but is unlikely to generate pairs of sites with both measures being high. When the site pairs of interest (Table 1) were overlaid on this null distribution, they could be seen to present both high scaled MI and PS values (red points) as expected from their high S-scores, except for pair 206/225 (green point).

From both the phylogenetic allelic structures (Fig. 2A) and the S-score rank 1 distribution (Fig. 2B), we concluded that six out of the seven site-pairs of interest were unlikely to have arisen by neutral evolutionary processes (496/503, 503/512, 451/485, 496/512, 439/451, 439/485). Even when looking at the distributions of rank 1 to 6 (Table S4), these pairs were found significantly outside their own simulated distributions (Fig. S8). In contrast, we were unable to reach the same conclusion for site-pair 206/225.

### Physical proximity may explain some of the non-overlap

**Figure 3:**
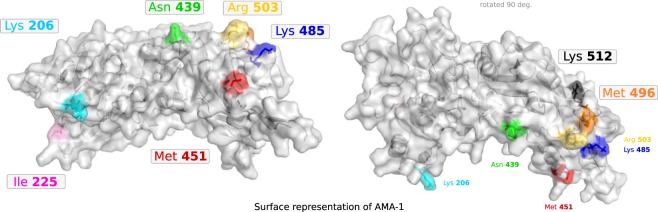
Surface representation of the crystal structure of AMA-1 with specific sites highlighted. The highlighted amino acids are the same in both panels and the ones relevant and described in Table 1 and Figs 1 and 2. The right panel is a 90 degree rotation of the left panel, in which Lys 512 and Met 496 are revealed. Amino acids are the consensus of all sequences.

Some of the pairs of sites which scored most highly on S-score and scaled MI comprised 496, 503 and 512. However, mapping these onto the tertiary structure of AMA-1 (Fig. 3) shows that they are in very close proximity of each other and therefore we were unable to exclude biochemical interactions as the primary cause of this striking association. It is still possible that variation at these sites is held in strong non-overlap through immune selection. Antibodies specific to epitopes containing amino acids I/N/K may cross-react with all other epitopic variants except those containing M/R/R, thereby promoting the co-circulation of these non-overlapping combinations of residues at these sites; however, because of their structural adjacency, it is impossible to privilege this explanation over intrinsic steric advantages to these combinations.

Other highly scored pairs included sites 439, 451, and 485, which were sufficiently far enough apart in the tertiary structure to represent potential examples of immune selection forcing non-structurally related residues to co-segregate into discordant sets. We cannot, of course, completely eliminate long-range physical interactions as a cause of these associations, and whether it is indeed immune selection that is responsible for these patterns can only be established by functional studies. There should be no fundamental structural impediment to the existence of intermediate forms since they do occur at low frequencies, but whether they are disadvantaged due to lower intrinsic fitness or immune-mediated selection remains to be resolved by further experimental work.

### Non-overlapping associations are also observed at the level of local populations

We also investigated the associations between sites 439, 451 and 485 on a smaller geographical scale to confirm that these patterns did not arise due to certain combinations of alleles dominating by chance in particular areas. In all countries studied, both of the major sequence variant associations were present, with the secondary variants present at a lower frequency (Table 2). In particular, the dataset of 660 unique sequences from Mali showed high non-overlap for each pair. A smaller dataset comprising 79 sequences from the Gambia also yielded scaled MI values in excess of those obtained from the global data set. Slightly weaker associations were found in 86 unique sequences from Kenya. However, these were seen to improve significantly when the data were analysed as a collection of isolates rather than sequences: i.e. without excluding identical sequences present in different individuals (Table 3).

**Table 2:** Country-specific counts of amino acid associations between positions 439, 451 and 485 within unique sequences obtained from The Gambia, Kenya and Mali.

Association	Country and Amino Acid Combinations					Scaled MI
439 vs 451		**HK**	**HM**	**NK**	**NM**	
	*Gambia*	46	3	0	31	0.701
	*Kenya*	46	3	9	27	0.402
	*Mali*	395	25	11	211	0.637
		**HI**	**HK**	**NI**	**NK**	
439 vs 485	*Gambia*	39	7	1	30	0.549
	*Kenya*	45	4	1	35	0.682
	*Mali*	359	61	17	205	0.453
451 vs 485		**KI**	**KK**	**MI**	**MK**	
	*Gambia*	39	4	1	35	0.673
	*Kenya*	46	9	0	31	0.515
	*Mali*	366	40	10	244	0.608

**Table 3:** Counts of amino acid associations between positions 439, 451 and 485 in a population study (duplicate sequences not excluded) in Kilifi, Kenya.

Association	Amino Acid Combinations				Scaled MI
439 vs 451	**HK**	**HM**	**NK**	**NM**	0.564
	61	3	9	55	
439 vs 485	**HI**	**HK**	**NI**	**NK**	0.772
	60	4	1	63	
451 vs 485	**KI**	**KK**	**MI**	**MK**	0.644
	61	9	0	59	

As the model predictions pertain to population level frequencies of allele combinations, rather than their presence in a collection of unique sequences, we believe that Table 3 presents a more valid test (than when only unique sequences are included) of the model’s capabilities to pick out regions under strong immune selection. It is nonetheless reassuring that these patterns are also evident under the more stringent conditions where only unique sequences are included.

## Discussion

In this study, we have set out to demonstrate reverse immunodynamics, a new population genetics approach that is able to identify targets of immune selection under the theoretical expectations of a well established, multi-locus framework of pathogen evolution. Our results suggest that DIII of AMA-1 contains several epitopes of lower variability that are under strong immune selection. These epitopes may play an important role in protective immunity alongside more polymorphic epitopes in the C1L region of DI which have so far compromised the development of an effective vaccine.

The identification of possible epitopes in DIII follows the conclusions of several studies based on standard population genetics (sPG) techniques that strongly indicate that DIII is under selection^34–36,44–47^. However, sPG such as Tajima’s D are generally applied to windows of amino acids, and have critically not been devised for, and do not have the theoretical ground to identify immunity as the source of selection^48,49^. Reverse immunodynamics complements sPG techniques by finely mapping loci, which are tentatively under immune pressure within regions known to be under selection (through sPG analyses, as is the case of DIII).

Much work has also been done previously on AMA-1 site-pairs using measures of linkage disequilibrium (LD) such as D or R-squared (e.g.^34–36^), demonstrating that the alleles of many pairs are less mixed than expected by random chance (even in the presence of high recombination rates). Typically, such signatures can be used to infer that selection pressure has structured alleles into non-random associations. While it is tempting to assume that site-pairs high on LD are also high on MI, this is not necessarily true. The key difference is that MI is both a measure of linkage and non-overlap, while LD ignores the latter (for empirical and theoretical demonstration see Supplementary Text 1). We set on to look for signatures of non-overlap as these are critically predicted to be driven by strong immune selection, and therefore LD was not suited for the analyses. Finally, AMA-1 is known to present high recombination potential^34–36^, giving rise to a wide range of haplotypes^35^. According to complex transmission models based on *strain theory*, non-overlap is still a self-emergent signature among antigenic loci even in the presence of high recombination rates^50^.

So far, the evidence that DIII elicits protective antibody responses is limited, but much research supports this possibility. For instance, at a functional level, DIII has been shown to bind to the erythrocyte membrane protein Kx^51^, and immune responses that interrupt this process may, therefore, have a significant inhibitory effect on parasite growth. Nair *et al*.^52^ have also shown that antibodies from a single blood donor affinity purified on DIII are inhibitory. In a study by Polley *et al*.^15^, it was found that antibody responses against recombinant full-length ectodomains were more strongly associated with protection than responses to those which did not express DIII, even though naturally acquired antibodies to recombinant DIII alone were rare. A virosomal formulation of loop I of DIII, comprising residues 446–490, has also been shown to elicit growth-inhibitory antibodies^53^ and volunteers within a vaccine trial containing this AMA-1 peptide exhibited both reduced blood stage growth rates and a higher rate of morphological parasite abnormalities^54^. A recent analysis of allele-specific efficacy of a vaccine against AMA-1^25^ showed that the relative risk ratio (RRR) of re-infection with strains containing vaccine-type amino acids among vaccinees vs controls was high (although not significant) at residues 439, 451 and 485. Interestingly, in the same study, the likelihood of a shift from a vaccine- to non-vaccine type residue among sequences obtained following vaccination was found to be significantly increased at position 485 and non-significantly increased at position 451.

An interesting feature of our analyses is that while particular groups of sites exhibit strong non-overlap, the associations between these groups appear to be random. For example, the triad 439–451–485 is in complete linkage equilibrium with triad 496–503–512. Furthermore, there are no non-overlapping associations present between the highly polymorphic residues in the C1L region of Domain I and the 439–451–485 triad (although the former exhibit strong non-overlap, Table S1). If our analyses are correct, then it would be sufficient to present the dominant non-overlapping variants of 439–451–485 (i.e. HKI and NMK) in a manner that induced strong immune responses to the epitopes containing these residues, rather than to try and cover the entire haplotypic diversity of AMA-1^55^. Immune responses to the epitopes containing residues 439, 451 and 485 appear to develop more slowly under natural conditions than antibodies to the highly polymorphic regions shielding the hydrophobic groove^15^ but should be easier to induce by vaccination than immune responses to completely conserved epitopes^56^.

Following theoretical expectations, our analyses emphasize that targets of natural immunity are not necessarily polarised between the completely invariant and the highly polymorphic, but also critically include a range of epitopes of intermediate variability that have so far been largely overlooked^3^. The *reverse immunodynamics* method introduced in this study has the potential to identify and direct research towards such targets. While the method is likely to evolve with its application to other datasets, we foresee that its future use will help solve the impasse reached in vaccine discovery and development of many multi-strain pathogens.

## Methods

### Genetic sequence selection

PfAMA-1 sequences were obtained by searching the NCBI Entrez nucleotide and protein databases. This yielded 2835 protein sequences and 2926 nucleotide sequences (up to and including year 2014). In cases when the GenBank nucleotide record did not include the corresponding protein sequence, these were translated using Biopython^57^ (v1.69).

The analysis was restricted to sequences between 400 and 622 amino acids in length. The lower limit aims to ensure that all the polymorphic regions across the three domains are included; the upper limit corresponds to the entire processed protein, and therefore removes any entries which contain the non-coding sequence. To avoid pseudoduplication, all identical sequences were removed, leaving 1198 unique sequences. These were then aligned using ClustalW^58^ (Clustal2, v2.1).

### Indels and other undetermined states

We first truncated our protein alignment to the region where we had >99% coverage. The resulting sequence alignment (from residue 149 to residue 534) had only 0.0025% of gaps (state ‘−’) and 0.0138% of undetermined amino acids (state ‘X’). In our analysis, we ignored all variants that had gap or undetermined alleles.

### Measuring non-overlap among dimorphic sites

A frequency analysis of all residues within the three domains was used to identify any polymorphic sites. Only variants with a frequency exceeding 2.5% were included in the subsequent analysis. This identified 44 dimorphic sites (Fig. S1). We searched for signatures of immune selection by analysing the mutual information (MI) scores of all pairs of sites (Table S1).

### Geographic sub-analysis

We repeated the calculation of MI for all isolates whose country of origin could be identified (either from the Entrez database or with reference to published papers).

### Phylogenetic analysis

The alignment was used to construct a maximum likelihood (ML) tree using Mega6^59^ under the JTT model (with also: 4 discrete categories of gamma-distributed rates among sites; the nearest-neighbour-interchange heuristic; and a very strong branch swap filter). The resulting ML tree was used to calculate the maximum parsimony score (PS) for each dimorphic site (Fig. S2; Table S1). PS was calculated using the Phangorn R-package^60^ (v2.3.1) function *parsimony* with default options. We defined a new measure herein termed S-score, as the product of the MI and the geometric mean of the PS for each site (values available in Table S2). We used Seq-Gen^61^ (v1.3.4) to simulate 1000 sets of protein sequences with the same phylogenetic characteristics, restricting variation at each of the sites to two amino acids.

### Tertiary structures

From the protein data bank (PDB) the sequences and tertiary structures of Plasmodium vivax AMA-1 (PvAMA-1, PDB 1W81) and PfAMA-1 domain III (PDB 1HN6) were downloaded. As the tertiary structure of 1HN6 was mostly unstructured, it could not be used on its own. Therefore a Fold and Function Assignment System (FFAS) was used to align the amino acid sequences of 1W81 and 1HN6. From this alignment a ‘hybrid’ structural model with 94% confidence was generated using SCWRL. This resulting model gave a single tertiary structure with the residues within domain III from PvAMA-1 (1W81) substituted by those from PfAMA-1 (1HN6) using the P. vivax structural coordinates. Pymol (http://www.pymol.org/) was used to visualise the ‘hybrid’ tertiary structure and to highlight the relevant residues.

## Supplementary information

Supplementary Text 1

Table S1

Table S2

Table S3

Table S4

## References

[1] von Delft, A. *et al*. The generation of a simian adenoviral vectored HCV vaccine encoding genetically conserved gene segments to target multiple HCV genotypes. *Vaccine*, 10.1016/j.vaccine.2017.10.079 (2017). 10.1016/j.vaccine.2017.10.079 PMC5756538 29203182

[2] Coughlan L (2018). Heterologous Two-Dose Vaccination with Simian Adenovirus and Poxvirus Vectors Elicits Long-Lasting Cellular Immunity to Influenza Virus A in Healthy Adults. EBioMedicine.

[3] Thompson CP (2018). A naturally protective epitope of limited variability as an influenza vaccine target. Nat. Commun..

[4] Gupta S (1996). The maintenance of strain structure in populations of recombining infectious agents. Nat. Med..

[5] Gupta S, Ferguson NM, Anderson RM (1998). Chaos, Persistence, and Evolution of Strain Structure in Antigenically Diverse Infectious Agents. Science (80-.)..

[6] Lourenço J, Wikramaratna PSPS, Gupta S (2015). MANTIS: an R package that simulates multilocus models of pathogen evolution. BMC Bioinformatics.

[7] Lourenço, J. *et al*. Lineage structure of Streptococcus pneumoniae may be driven by immune selection on the groEL heat-shock protein. *Sci*. *Rep*. **7**, (2017). 10.1038/s41598-017-08990-z PMC5567354 28831154

[8] Hinnebusch J, Barbour AG, Restrepo BI, Schwan TG (1998). Population structure of the relapsing fever spirochete Borrelia hermsii as indicated by polymorphism of two multigene families that encode immunogenic outer surface lipoproteins. Infect. Immun..

[9] Johnson DR, Kaplan EL, VanGheem A, Facklam RR, Beall B (2006). Characterization of group A streptococci (Streptococcus pyogenes): correlation of M-protein and emm-gene type with T-protein agglutination pattern and serum opacity factor. J. Med. Microbiol..

[10] Areschoug T, Stålhammar-carlemalm M, Larsson C, Lindahl G (1999). Group B Streptococcal Surface Proteins as Targets for Protective Antibodies: Identification of Two Novel Proteins in Strains of Serotype V Group B Streptococcal Surface Proteins as Targets for Protective Antibodies: Identification of Two Novel. Proteins..

[11] Buckee CO, Gupta S, Kriz P, Maiden MCJJ, Jolley KA (2010). Long-term evolution of antigen repertoires among carried meningococci. Proc. Biol. Sci..

[12] Callaghan MJ (2008). The effect of immune selection on the structure of the meningococcal opa protein repertoire. PLoS Pathog..

[13] Lourenço J, Recker M (2016). Dengue serotype immune-interactions and their consequences for vaccine impact predictions. Epidemics.

[14] Remarque EJ, Faber BW, Kocken CHM, Thomas AW (2008). Apical membrane antigen 1: a malaria vaccine candidate in review. Trends Parasitol..

[15] Polley SD (2004). Human antibodies to recombinant protein constructs of Plasmodium falciparum Apical Membrane Antigen 1 (AMA1) and their associations with protection from malaria. Vaccine.

[16] Fowkes, F. J. I., Richards, J. S., Simpson, J. A. & Beeson, J. G. The relationship between anti-merozoite antibodies and incidence of Plasmodium falciparum malaria: A systematic review and meta-analysis. *PLoS Med*. **7** (2010). 10.1371/journal.pmed.1000218 PMC2808214 20098724

[17] Richards JS (2013). Identification and Prioritization of Merozoite Antigens as Targets of Protective Human Immunity to Plasmodium falciparum Malaria for Vaccine and Biomarker Development. J. Immunol..

[18] Mugyenyi, C. K. *et al*. Antibodies to Polymorphic Invasion-Inhibitory and Non-Inhibitory Epitopes of Plasmodium falciparum Apical Membrane Antigen 1 in Human Malaria. *PLoS One* **8** (2013). 10.1371/journal.pone.0068304 PMC3702562 23861883

[19] Stanisic DI (2015). Acquisition of antibodies against Plasmodium falciparum merozoites and malaria immunity in young children and the influence of age, force of infection, and magnitude of response. Infect. Immun..

[20] Osier, F. H. *et al*. Malaria: New antigens for a multicomponent blood-stage malaria vaccine. *Sci*. *Transl*. *Med*. **6** (2014). 10.1126/scitranslmed.3008705 PMC4856877 25080477

[21] Muh F (2018). *In vitro* invasion inhibition assay using antibodies against Plasmodium knowlesi Duffy binding protein alpha and apical membrane antigen protein 1 in human erythrocyte-adapted P. knowlesi A1-H.1 strain. Malar. J..

[22] Vicentin EC (2014). Invasion-inhibitory antibodies elicited by immunization with Plasmodium vivax apical membrane antigen-1 expressed in Pichia pastoris yeast. Infect. Immun..

[23] Biswas S (2012). Recombinant Viral-Vectored Vaccines Expressing Plasmodium chabaudi AS Apical Membrane Antigen 1: Mechanisms of Vaccine-Induced Blood-Stage Protection. J. Immunol..

[24] Schussek S (2013). Immunization with apical membrane antigen 1 confers sterile infection-blocking immunity against plasmodium sporozoite challenge in a rodent model. Infect. Immun..

[25] Ouattara A (2013). Molecular basis of allele-specific efficacy of a blood-stage malaria vaccine: Vaccine development implications. J. Infect. Dis..

[26] Drew, D. R. *et al*. Defining the Antigenic Diversity of Plasmodium falciparum Apical Membrane Antigen 1 and the Requirements for a Multi-Allele Vaccine against Malaria. *PLoS One* **7** (2012). 10.1371/journal.pone.0051023 PMC3515520 23227229

[27] Dicko A (2007). Impact of a plasmodium falciparum AMA1 vaccine on antibody responses in adult Malians. PLoS One.

[28] Thera, M. A. *et al*. Safety and immunogenicity of an AMA-1 malaria vaccine in Malian adults: Results of a phase 1 randomized controlled trial. *PLoS One* **3** (2008). 10.1371/journal.pone.0001465 PMC2186380 18213374

[29] Laurens, M. B. *et al*. Extended safety, immunogenicity and efficacy of a blood-stage malaria vaccine in Malian children: 24-Month follow-up of a randomized, double-blinded phase 2 trial. *PLoS One* **8** (2013). 10.1371/journal.pone.0079323 PMC3832522 24260195

[30] Biswas, S. *et al*. Assessment of humoral immune responses to blood-stage malaria antigens following ChAd63-MVA immunization, controlled human malaria infection and natural exposure. *PLoS One* **9** (2014). 10.1371/journal.pone.0107903 PMC4177865 25254500

[31] Bai T (2005). Structure of AMA1 from Plasmodium falciparum reveals a clustering of polymorphisms that surround a conserved hydrophobic pocket. Proc. Natl. Acad. Sci..

[32] Lim SS (2014). Structure and dynamics of apical membrane antigen 1 from plasmodium falciparum FVO. Biochemistry.

[33] Crosnier C (2016). Binding of plasmodium falciparum merozoite surface proteins DBLMSP and DBLMSP2 to human immunoglobulin M Is conserved among broadly diverged sequence variants. J. Biol. Chem..

[34] Polley SD, Conway DJ (2001). Strong diversifying selection on domains of the Plasmodium falciparum apical membrane antigen 1 gene. Genetics.

[35] Lumkul, L., Sawaswong, V., Simpalipan, P. & Kaewthamasorn, M. Unraveling Haplotype Diversity of the Apical Membrane Antigen-1 Gene in Plasmodium falciparum Populations in Thailand. **56**, 153–165 (2018). 10.3347/kjp.2018.56.2.153 PMC5976018 29742870

[36] Kang J-M (2018). Population genetic structure and natural selection of Plasmodium falciparum apical membrane antigen-1 in Myanmar isolates. Malar. J..

[37] Richard D (2010). Interaction between Plasmodium falciparum apical membrane antigen 1 and the rhoptry neck protein complex defines a key step in the erythrocyte invasion process of malaria parasites. J. Biol. Chem..

[38] Lamarque, M. *et al*. The RON2-AMA1 interaction is a critical step in moving junction-dependent invasion by apicomplexan parasites. *PLoS Pathog*. **7**, (2011). 10.1371/journal.ppat.1001276 PMC3037350 21347343

[39] Srinivasan P (2011). Binding of Plasmodium merozoite proteins RON2 and AMA1 triggers commitment to invasion. Proc. Natl. Acad. Sci..

[40] Healer J (2004). Allelic polymorphisms in apical membrane antigen-1 are responsible for evasion of antibody-mediated inhibition in Plasmodium falciparum. Mol. Microbiol..

[41] Cortés A (2005). Allele Specificity of Naturally Acquired Antibody Responses against Plasmodium falciparum Apical Membrane Antigen 1. Society.

[42] Tanabe K, Mackay M, Goman M, Scaife JG (1987). Allelic dimorphism in a surface antigen gene of the malaria parasite Plasmodium falciparum. J. Mol. Biol..

[43] Marshall VM (1991). A Plasmodium falciparum MSA-2 gene apparently generated by intragenic recombination between the two allelic families. Mol. Biochem. Parasitol..

[44] Arnott A (2014). Distinct patterns of diversity, population structure and evolution in the AMA1 genes of sympatric Plasmodium falciparum and Plasmodium vivax populations of Papua New Guinea from an area of similarly high transmission. Malar J.

[45] Amambua-Ngwa, A. *et al*. Population Genomic Scan for Candidate Signatures of Balancing Selection to Guide Antigen Characterization in Malaria Parasites. *PLoS Genet*. **8** (2012). 10.1371/journal.pgen.1002992 PMC3486833 23133397

[46] Osier FHA (2010). Allelic diversity and naturally acquired allele-specific antibody responses to Plasmodium falciparum apical membrane antigen 1 in Kenya. Infect. Immun..

[47] Tetteh, K. K. A. *et al*. Prospective identification of malaria parasite genes under balancing selection. *PLoS One* **4** (2009). 10.1371/journal.pone.0005568 PMC2679211 19440377

[48] Pavlidis P, Alachiotis N (2017). A survey of methods and tools to detect recent and strong positive selection. J. Biol. Res..

[49] Bhatt S, Katzourakis A, Pybus OG (2010). Detecting natural selection in RNA virus populations using sequence summary statistics. Infect. Genet. Evol..

[50] Buckee CO, Recker M, Watkins ER, Gupta S (2011). Role of stochastic processes in maintaining discrete strain structure in antigenically diverse pathogen populations. Proc. Natl. Acad. Sci. USA.

[51] Kato K, Mayer DCG, Singh S, Reid M, Miller LH (2005). Domain III of Plasmodium falciparum apical membrane antigen 1 binds to the erythrocyte membrane protein Kx. Proc. Natl. Acad. Sci. USA.

[52] Nair M (2002). Structure of domain III of the blood-stage malaria vaccine candidate, Plasmodium falciparum apical membrane antigen 1 (AMA1). J. Mol. Biol..

[53] Mueller MS (2003). Induction of parasite growth-inhibitory antibodies by a virosomal formulation of a peptidomimetic of loop I from domain III of Plasmodium falciparum apical membrane antigen 1. Infect. Immun..

[54] Thompson, F. M. *et al*. Evidence of blood stage efficacy with a virosomal malaria vaccine in a phase IIa clinical trial. *PLoS One* **3** (2008). 10.1371/journal.pone.0001493 PMC2204057 18231580

[55] Duan J (2008). Population structure of the genes encoding the polymorphic Plasmodium falciparum apical membrane antigen 1: implications for vaccine design. Proc. Natl. Acad. Sci. USA.

[56] Dutta S (2013). Overcoming Antigenic Diversity by Enhancing the Immunogenicity of Conserved Epitopes on the Malaria Vaccine Candidate Apical Membrane Antigen-1. PLoS Pathog..

[57] Cock PJA (2009). Biopython: freely available Python tools for computational molecular biology and bioinformatics. Bioinformatics.

[58] Larkin MA (2007). Clustal W and Clustal X version 2.0. Bioinformatics.

[59] Tamura K, Stecher G, Peterson D, Filipski A, Kumar S (2013). MEGA6: Molecular Evolutionary Genetics Analysis version 6.0. Mol. Biol. Evol..

[60] Schliep K (2011). P. phangorn: Phylogenetic analysis in R. Bioinformatics.

[61] Rambaut A, Grassly NC (1997). Seq-Gen: an application for the Monte Carlo simulation of DNA sequence evolution along phylogenetic trees. Mol. Biol..

