# Supplementary material for: Reverse immunodynamics: a new method for identifying targets of protective immunity
Source: Sci Rep. 2019 Feb 15;9:2164. doi: 10.1038/s41598-018-37288-x (PMC6377634; doi:10.1038/s41598-018-37288-x)
Supplement: Supplementary file 1 — Supplementary Text 1 [file 41598_2018_37288_MOESM1_ESM.doc]

Supplementary Material:

full methods and additional figures

Reverse immunodynamics: a new method for identifying targets of protective immunity

Katrina J Spensley**^a*^**, Paul S Wikramaratna**^b*^**, Bridget S Penman**^c^**, Andrew Walker**^c^**, Adrian L Smith**^c^**, Oliver G Pybus**^c^**, Létitia Jean**^d^**, Sunetra Gupta**^c^** & José Lourenço**^c**^**

a. Imperial College London, London W2 1PG

b. Institute of Evolutionary Biology, University of Edinburgh, Edinburgh EH9 3JT

c. Department of Zoology, University of Oxford, Oxford OX1 3PS

d. The Sir William Dunn School of Pathology, University of Oxford, Oxford OX1 3RE

*these authors contributed equally to the manuscript

**Correspondence to: **jose.lourenco@zoo.ox.ac.uk**

Table of Contents

[*Strain theory* predictions 2](#__RefHeading___Toc191_682284829)

[Details on methods used 3](#__RefHeading___Toc367_260106886)

[Mutual information 3](#__RefHeading___Toc193_682284829)

[Scaled mutual information 4](#__RefHeading___Toc2041_1657436870)

[Linkage disequilibrium 4](#__RefHeading___Toc195_682284829)

[Calculation of MI, LD and their variants 5](#__RefHeading___Toc27319_2846039045)

[Parsimony score 5](#__RefHeading___Toc365_260106886)

[Linkage disequilibrium versus mutual information 6](#__RefHeading___Toc186_682284829)

[Empirical pairs of sites 6](#__RefHeading___Toc3210_1652704675)

[*In silico* pairs of sites 8](#__RefHeading___Toc3212_1652704675)

[Other supplementary figures 12](#__RefHeading___Toc188_682284829)

[References 16](#__RefHeading___Toc2757_858959509)

# *Strain theory* predictions

The role of immune selection in driving pathogen dynamics has been well studied within theoretical frameworks [1]–[8]⁠⁠. One of such frameworks, introduced by Gupta and colleagues and herein termed as *strain theory* [2]⁠, proposes the existence of three universal and discrete dynamic behaviours for multi-strain pathogen systems (termed *strain structures*).

In summary, a host can cross-recognize shared antigenic alleles in different genetic variants of the same pathogen. This recognition leads to cross-immunity, assumed to restrict the future within-host infection potential of variants carrying antigenic alleles already experienced. The strength of cross-immunity (acquired indirectly by the host to so far inexperienced variants) dictates the level of competition between circulating variants, and influences the resulting population dynamics (termed *strain structures*).

When antigenic alleles induce little or no cross-immunity between variants, *no strain structure* (NSS) is predicted, in which antigenic variants circulate seemingly independently at very similar prevalences due to lack of competition. If instead intermediate-to-high cross-immunity is induced, *cyclic strain structure* (CSS) is predicted, because competition for susceptible hosts desynchronizes the population dynamics of the antigenic variants leading to cyclical variant dominance over time (e.g. Influenza A viruses at the population level [9]–[11]⁠, or within-host *P. falciparum [12]*⁠ and HIV [13]⁠). Finally, if very high cross-immunity is induced, competitive exclusion is predicted to occur between antigenic types sharing alleles. This *strain structure* is termed *discrete strain structure* (DSS), since extant variants are predicted to express non-overlapping combinations of antigenic alleles to avoid competition and therefore extinction (e.g. PorA and FetA surface antigens of *N. meningitidis* [12], [14]–[16]⁠, dengue virus serotypes [6], [17]⁠, or capsular genes and others of *S. pneumoniae* [18], [19]⁠).


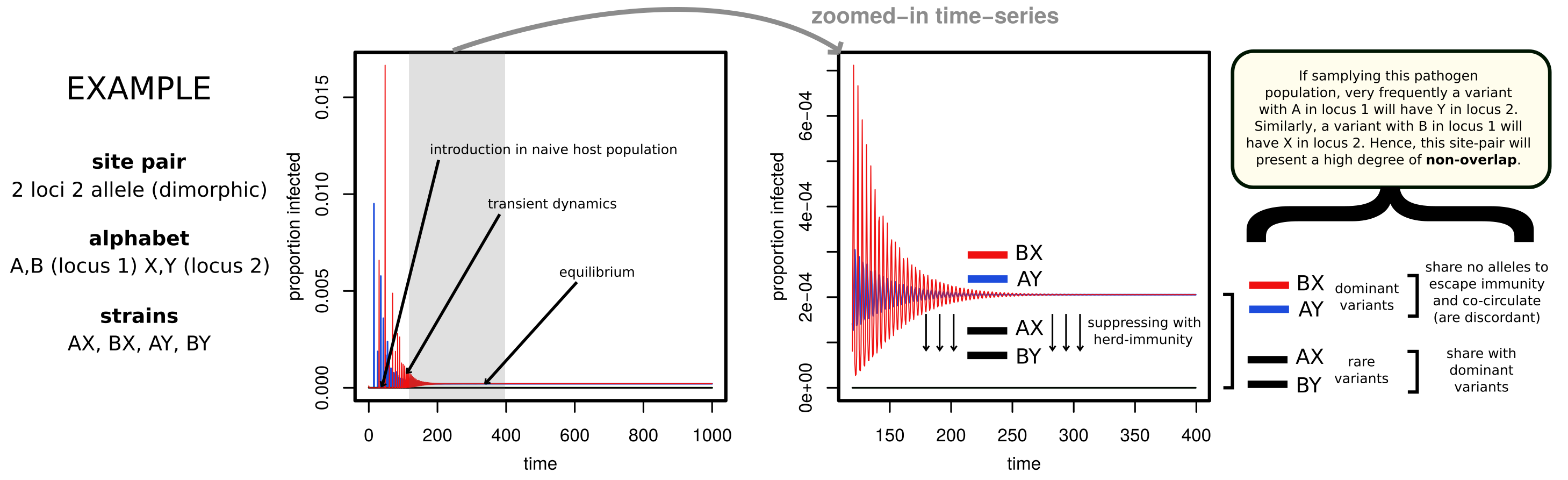
A simple example of the transmission and immune selection of a dimorphic pair of sites (a 2 loci 2 allele) is simulated using the MANTIS R-package [8]⁠. MANTIS is a simulator of the basic model behind *strain theory*, and for this example cross-immunity (parameter gamma) was set to 0.99 and the basic reproductive number was 2. At equilibrium, strong immune selection is seen to result in competitive exclusion between antigenic loci (locus 1 and 2), such that the variants self-organize into discrete strain structure (DSS). As a result, if the pathogen population were to be sampled at equilibrium, it would be very likely that 2 variants would be frequent and 2 variants would be infrequent. The frequent variants would show strong signatures of non-overlap, as selection would have dictated that co-circulation was dependent on sharing as little antigenic alleles as possible.

In this study, we are interested in using *strain theory’s* predictions to identify particular amino acid sites under strong immune selection. We therefore focus on the theoretical expectation that such loci should self-organize into a discrete strain structure (DSS) in which circulating variants present a high degree of non-overlap.

# Details on methods used

### Mutual information

Mutual Information (MI) is a measure of the mutual dependence of two variables [20]⁠ that has been previously applied to quantify co-evolution between nucleotide or amino acid sites in multiple sequence alignments [21]–[23]⁠. When applied to sequence data, MI captures how much information can be gained about one site from another, and is a function of the entropy of the two sites, their joint entropy and / or their conditional entropy.

Shannon’s entropy, *H(X)*, is a measure of the uncertainty of a discrete random variable, *X*. The possible values of *X* are defined by an alphabet $K=\left\{x_{1},x_{2},\ldots,x_{k}\right\}$, with each letter having a probability of being observed $p\left(x\right)=\left\{p\left(x_{1}\right),p\left(x_{2}\right),\ldots,p\left(x_{k}\right)\right\}$. If we set the random variable *X* to be an amino acid site in a sequence, then the entropy is defined as the sum of the product of the probability and log probability of the amino acids (letters) in that site:

$$H\left(X\right)=-\sum_{i=1}^{k} p\left(x_{i}\right)\text{log}_{b}p\left(x_{i}\right)$$

Here, *b* is a scalar of entropy, commonly the exponential (used in our results). It is important to note that *H(X)* is a common diversity measure in ecology (termed *Shannon index*), which is maximised when all K letters (species in ecology, or amino acids in this study) are present in equal quantity and minimised when only one of the possible letters is present.

The joint entropy of two sites, *H(X,Y),* follows a similar formulation, but taking into account their joint alphabet. In a simple example, if site *X* has letters *K= {A,B}* and site *Y* has letters *l= {R,S}*, the full and possible joint alphabet of XY is *m= {AR, AS, BR, BS},* with each member also having a probability of being observed. The joint entropy is defined as the sum of the product of the probability and log probability of each member in *m* of that pair of sites:

$$H\left(X,Y\right)=-\sum_{i=1}^{k} \sum_{j=1}^{l} p\left(x_{i},y_{j}\right)\text{log}_{b}p\left(x_{i},y_{j}\right)$$

The conditional entropy *H(X|Y)* is the entropy of *X* given *Y*, or *H(Y|X)* the entropy of *Y* given *X,* formally defined as:

$$H\langle X|Y\rangle=H\left(X,Y\right)-H\left(Y\right)$$

$$H\langle Y|X\rangle=H\left(Y,X\right)-H\left(X\right)$$

The mutual information of the pair of sites, *MI(X,Y)*, can be obtained from one of the following three formulations, as visualised in the diagram below. Note that *MI(Y,X) = MI(X,Y)*.

*MI(X,Y) = H(X) – H(X|Y)*

*MI(X,Y) = H(Y) – H(Y|X)*

*MI(X,Y) = H(X) + H(Y) – H(X,Y)*


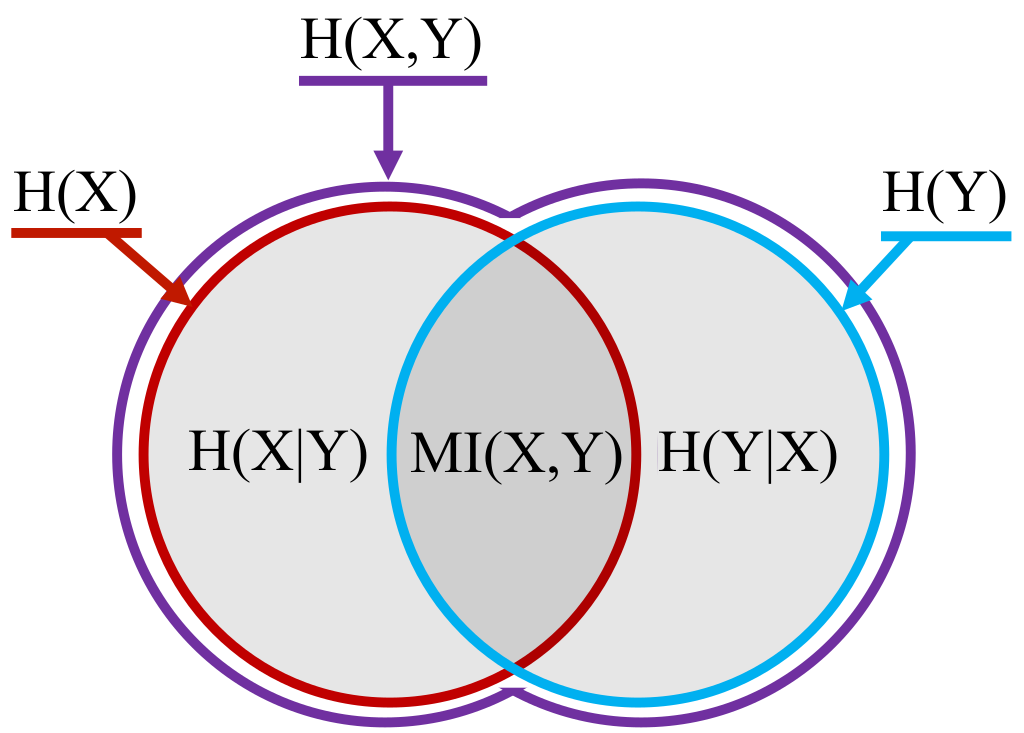


### Scaled mutual information

Shannon’s entropy (or diversity index), *H(X)*, is a positive number, with maximum value equal to *log_b_(k)*. When looking at a particular amino acid site *X, k* is the number of observed (unique) amino acids in *X*. Similarly, when looking at pairs of sites sites *XY*, then *k* is the number of observed (unique) amino acid pairs in *XY*. In the simple example described above, if site X has letters *K= {A,B}* and site Y has letters *l= {R,S}*, the full and possible joint alphabet is *m= {AR, AS, BR, BS}* with *k*=4. Along a genetic sequence, amino acid diversity varies. The interpretation of entropy (or diversity index) between two sites with different *k* is problematic, and is common to normalise (scale) entropy by its maximum value.

The same issue applies to mutual information of a pair of sites, *MI(X,Y)*, for which the absolute scale will be dependent on the joint alphabet of XY. For an alphabet of size *i* for site *X*, and alphabet of size *j* for site *Y*, the maximum *MI(X,Y)* is known to be *log_b_(min{i,j})*. In this study we present both the absolute MI values and scaled MI values by dividing by *log_b_(min{i,j})*. In the main results, we look at dimorphic sites and therefore divide MI by l*og_e_(2).* We interpret the main results using the scaled MI alone.

### Linkage disequilibrium

Linkage disequilibrium (LD) measures are commonly used to describe the associations of (genetic) alleles [24], [25]⁠, and can be interpreted and formulated as the covariance (COV) between two random variables *X* and *Y* which are loci (sites) on genetic sequences (*X* and *Y* being variables like the ones defined for MI). For example, if we take the presence for two possible amino acids A and B at one locus (site *X*) and R and S at another (site *Y*), we define the possible joint alphabet of XY as *m= {AR, AS, BR, BS},* and the following frequency (probability) table:

| X \ Y | **R** | **S** | marginal |
| --- | --- | --- | --- |
| **A** | *x1* | *x2* | *p1* |
| **B** | *x3* | *x4* | *q1* |
| marginal | *p2* | *q2* |  |

where, for instance, the combination of alleles BR is observed with frequency (or probability) *x3, with* *q1* the frequency (or probability) of B, and *p2* the frequency (or probability) of R.

The association between B and R is taken as random when the occurrence of B does not affect the occurrence of R (and vice-versa). In such a case, B and R are independent and the probability of their co-occurrence is *x3=q1.p2*. It is taken as *disequilibrium* when *x3* diverges from *q1.p2.* Hence, linkage (D) between B and R can be measured by:

*D_BR_= COV{B,R}= x3 – q1.p2*

It should be noted that *D_BR_* refers to particular amino acids and not the sites themselves. Other combinations of amino acids on the same sites may present different D because their frequencies will be different. In other words, D as a coefficient of disequilibrium makes it difficult to compare loci and / or alleles within loci. It is therefore common to use scaled (or normalised) LD measures such as D’ or R^2^ which represent the loci instead of particular alleles [26]⁠. In summary, the measure D’ still retains some issues, such as being more likely to take extreme values when frequencies are small; in this study we therefore use R^2^, which can be calculated by:

*R^2^ = D_ij_^2^ / p_i_.(1-p_i_).p_j_.(1-p_j_)*

In the example above of the pair B and R, linkage disequilibrium of the sites X and Y would therefore be calculated as:

*R^2^ = D_BR_^2^ / q1.(1-q1).p2.(1-p2)*

*R^2^* varies between 0 and 1, with 0 meaning no linkage and 1 maximum linkage.

*

*

### Calculation of MI, LD and their variants

In this study no particular software or algorithm was used to calculate MI and LD. Measures were calculated using R, in which the above mathematical formulations were applied per pair of sites.

### Parsimony score

The parsimony score (PS) of an amino acid site *X* indicates the minimum number of estimated genetic changes at *X* under a phylogenetic tree derived from all sites. Hence, PS measures how much change was required in a particular site to observe the ancestral relationships of the samples under study. In our study we focus on pairs of sites, and therefore calculated PS of a pair by computing the geometric mean of the two independent parsimony scores (GMPS) of each site.

In practice, if a pair of sites has high GMPS, then multiple independent and convergent paired amino acid changes have occurred along the phylogenetic tree. In contrast, a low value implies rare independent and convergent paired amino acid changes along the phylogenetic tree. The first is exceptionally unlikely to be observed in the absence of selection, while the second can be observed more easily (e.g. clonal expansion not related to selection, population structure, ancestry, etc).

In the main text, we use measures of GMPS as an heuristic to assess which pairs of sites with high mutual information we can not exclude the possibility of neutral processes as the main driver (as opposed to selective processes).

# Linkage disequilibrium versus mutual information

In the main text we present and interpret our result based on scaled mutual information (MI) and parsimony scores of a set of dimorphic pairs of sites. Here we present linkage disequilibrium (LD) against MI of those pairs of sites, making a case for why LD is less ideal to identify the particular genetic signatures induced by strong immune pressure - as predicted by *strain theory*.

### Empirical pairs of sites

We started by characterising some useful thresholds of observed distributions of the dimorphic sites in the main text (Figure S1). We found that 95% of the pairs of sites were at a distance of 3 to 321 positions in sequence space (Figure S1A). When measuring linkage and mutual information, we found that 95% of the pairs of sites had LD between 0.0000315 and 0.286 and MI between 0.0000228 and 0.205 (Figures S1BC).

These 95% confidence intervals (CIs) had different uses. For the distance CI, in the context of the results presented in the main text, we used the lower bound of 3 positions (Figure S1A) to ignore any pair of sites with high MI at 3 positions away or less (since these were statistical outliers given the 95% CI). For the LD and MI CIs, in the context of the results presented in this supplementary material, we used the upper bounds to identify the subset of pairs of sites which are seen to have significantly higher LD or / and MI than expected from the observed distribution (Figure S2).

Please note that in the main text we used a more stringent upper bound for MI, from the 98% CI instead.


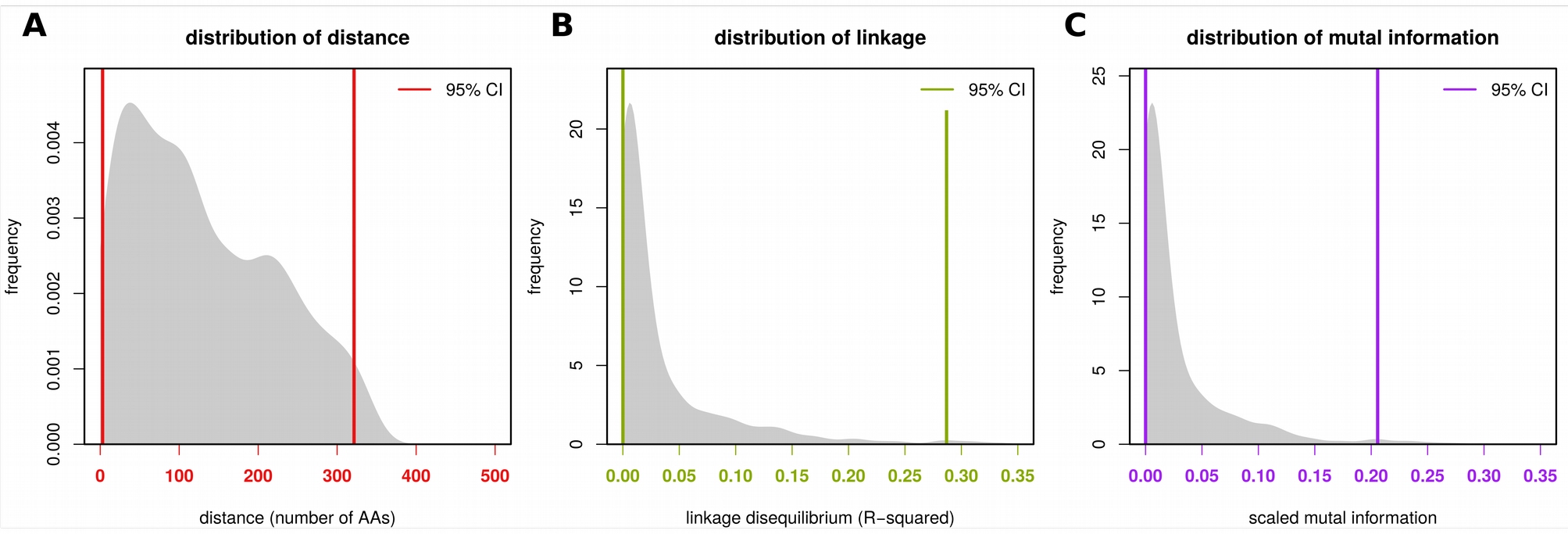

**Figure S1 – observed distributions and confidence intervals of site pair distance, linkage disequilibrium and mutual information.** Subplots A, B and C present information related to the dimorphic pairs of sites of the main results (see Figure 1), available in Table S2. **(A)** Distribution of distance in sequence space of the two sites in each pair. The red lines mark the 95% confidence interval at 3 and 321. **(B)** Distribution of linkage disequilibrium values (R^2^) for each pair. The green lines mark the 95% confidence interval at 0.0000315 and 0.286. **(C)** Distribution of mutual information (scaled) for each pair. The purple lines mark the 95% confidence interval at 0.0000228 and 0.205.

Figure S2 presents the LD and MI values for all dimorphic pairs of sites (N=946, same sites as in main results, Figure 1 and Table S2). Applying the lower bound for distance (=3) based on the observed 95% CI of distances between pairs resulted in the rejection of 2.75% of the pairs of sites (in red across all subplots of Figure S2). Notably, the percent of pairs of sites above the LD (Figure S2A) and MI (Figure S2B) upper bounds based on the observed 95% CIs was the same, leading to a rejection of 97.46% of the pairs. Figure S2C shows that while the seven pairs discussed in detail in the main text would be selected by both LD and MI thresholds (cyan points), LD was consistently higher than MI for all pairs even tho both measures were normalised between 0 and 1. Again, it should be reminded that in the main text we used a more stringent upper bound for MI instead (98.5% CI), which is how the cyan pairs where selected.


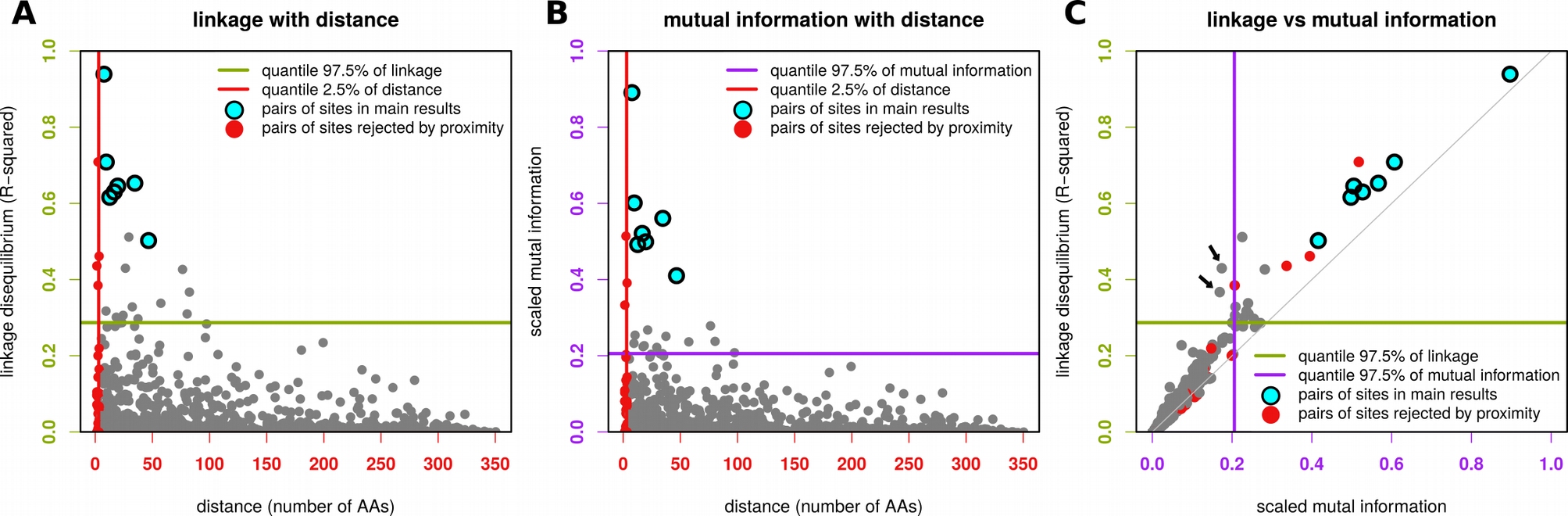

**Figure S2 – Comparison of observed site pair linkage disequilibrium and mutual information.** Subplots A, B and C present information related to the dimorphic pairs of sites of the main results (see Figure 1), available in Table S2. In all subplots, the red points are the pairs which are 3 or less positions away from one another (red vertical line, see description of Figure S1); the cyan points mark the seven site pairs that are part of the main results (see Figure 1). **(A)** Linkage disequilibrium (LD, R^2^) for all pairs according to distance (number of positions) between the two sites of the pair. The green horizontal line marks the upper limit of the 95% confidence interval of the observed LD values. **(B)** Mutual information (scaled MI) for all pairs according to distance (number of positions) between the two sites of the pair. The purple horizontal line marks the upper limit of the 95% confidence interval of the observed MI values. **(C)** MI versus LD for all pairs. The grey line is the diagonal, in which points would be if LD=MI. The green horizontal, and purple vertical lines mark the upper limit of the 95% confidence interval of the observed LD and MI values, respectively.

As show by the two examples marked with black arrows (Figure S2C), there would be pairs of sites selected by LD but not MI. In fact, two sets of four pairs would be selected by one measure but not the other: four pairs would be selected with the 95% CI of LD but not MI (positions 19-42, 182-184, 177-259, 25-51); four other pairs would be selected with the 95% CI of MI but not LD (positions 14-24, 160-257, 257-291, 77-94). Figure S3 shows in detail the set of pairs which would be selected by LD alone.

From these LD selected pairs, three out of four showed an immediately clear common characteristic – they were dominated by one of the four possible alleles, with it reaching 88%, 86% and 82% prevalence across the samples (these pairs are highlighted in Figure S3 as (a), (b), (c), respectively). The fourth pair had a weaker signal but still presented dominance of one allele at 65% (marked with (d) in Figure S3). It is thus not surprising that LD was high for these site pairs, given that a dominance of one of the alleles indicates that a particular amino acid in one site is very often observed along another particular amino acid in the other site. For instance, for site pair (a), amino acid N on one site appears 88% of the times with amino acid R on the other site. This type of information offered by one site on another misses a fundamental property of *linkage* predicted by *strain theory* – that pairs of sites under strong immune selection should self-organize into non-overlapping combinations of alleles; while what is observed here is the simple dominance of one allele, e.g. N R in pair (a). In the next section we explore the differences between the type of linkage measured by LD and MI through simulation.

| 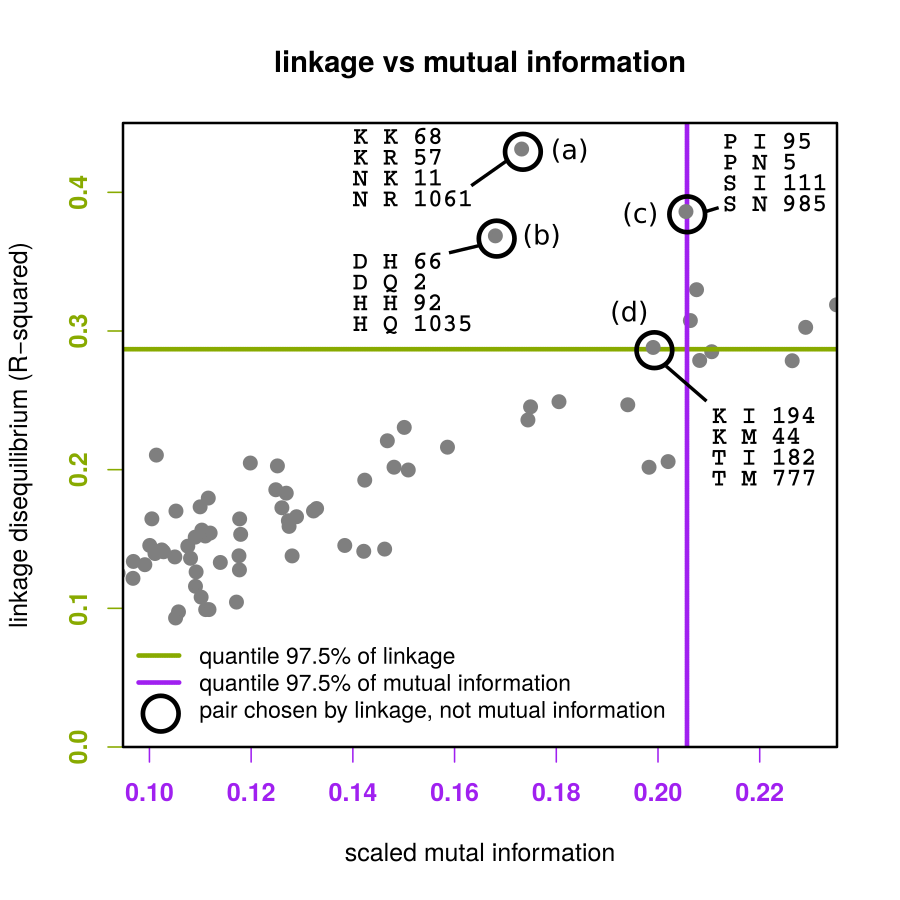 | **Figure S3 – Details on the set of site pairs that would be selected using linkage disequilibrium but not mutual information.**  This figure is a zoom-in representation of Figure S2C, trimming the x-axis between 0.10 and 0.23, and the y-axis between 0 and 0.45. The four site pairs that would be chosen as relevant for signatures of immune selection by linkage disequilibrium (LD) alone are marked with black empty circles; also labelled with (a), (b), (c) and (d). The observed dimorphic, amino acid allele counts for each pair is represented close to each respective point. The horizontal green line marks the upper bound of the 95% confidence interval for LD. The vertical purple line marks the upper bound of the 95% confidence interval for mutual information. |
| --- | --- |

### *In silico* pairs of sites

To further understand and describe the type of *linkage* measured by LD and MI, we resorted to simulation. We calculated these measures over 16000 simulated pairs of dimorphic sites with allele counts under different distributions. For this, we used the alphabets *I={R,S}* and *J={X,Y}*, having a joint alphabet *K={RY, RX, SY, SX}*, sampled 1000 different counts for each allele of *K* (counts in 1 to 1000), and applied 16 different types of masks to each of the 1000 counts (as illustrated below). With the 16 masks we effectively generated pairs of sites under all possible levels of count asymmetry.


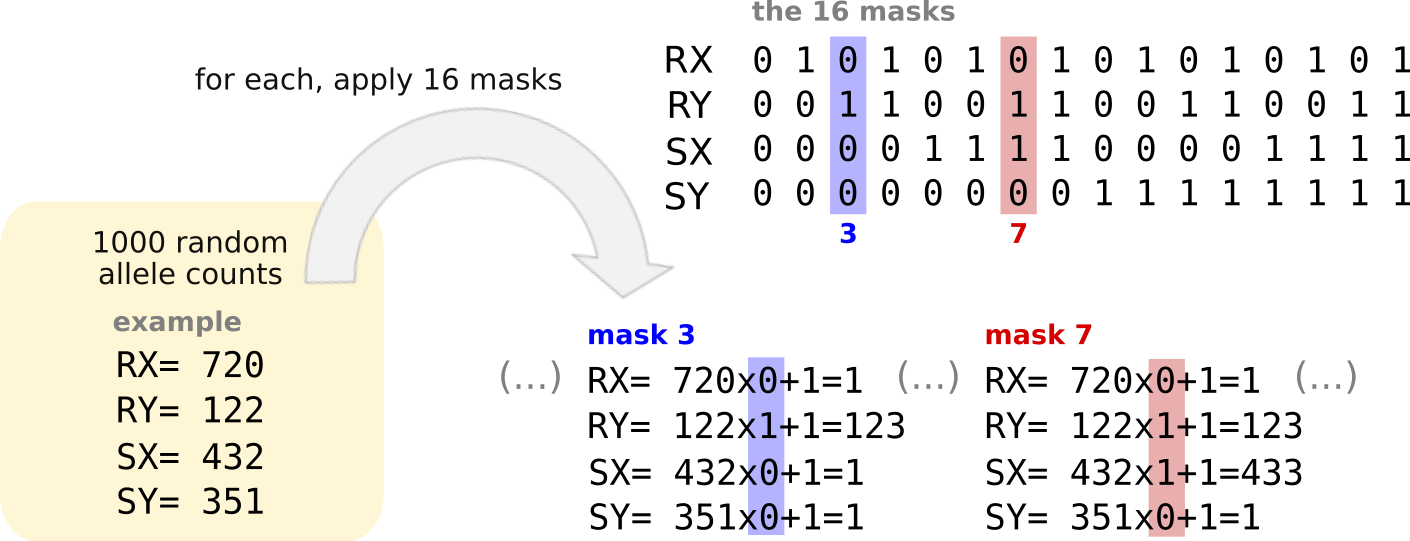


The results of this *in silico* experiment are presented in Figure S4, in which we coloured each site pair according to how many of their possible four alleles were set to one count (the minimum) under the 16 masks described above (i.e. when masks made these alleles extremely rare). We here describe the results by focusing on the four possible subsets of pairs: the ones that had three out of four alleles set to minimum (coloured in red), the ones that had two out of four alleles set to minimum (coloured in blue), the ones that had one out of four alleles set to minimum (coloured in green), and the ones which were purely random (coloured in grey).


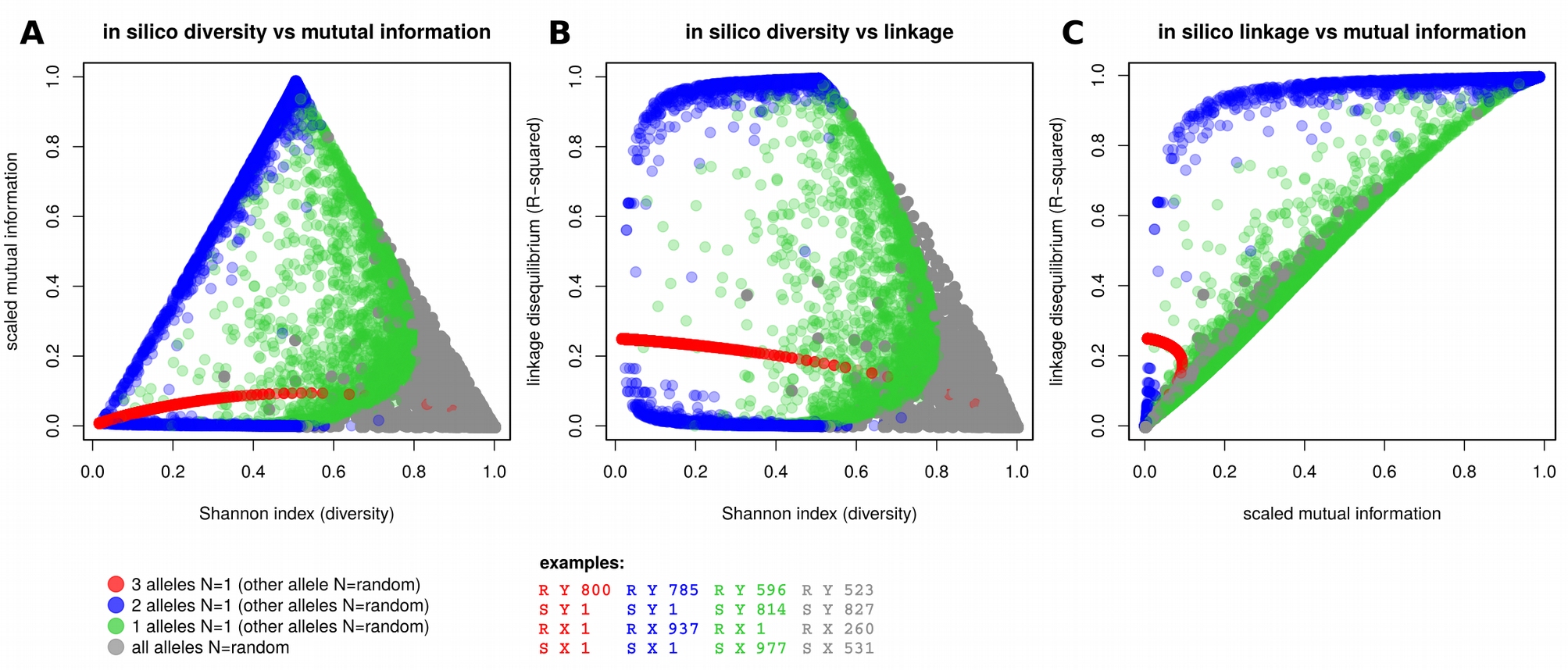

**Figure S4 - measures of linkage disequilibrium and mutual information for *in silico* pairs of sites.** Subplots A, B, C show mutual information, linkage disequilibrium and diversity results for 16000 simulated pairs of dimorphic sites with allele counts under different distributions (see details in the text). Four types of allele combinations and counts are considered (red, blue, green, grey). Examples of the types of allele combinations and counts are presented in the figure legend. **(A)** Shannon Index (diversity) against mutual information (scaled, MI). **(B)** Shannon Index (diversity) against linkage disequilibrium (LD, R^2^). **(C)** LD against MI.

For site pairs in which three out of four alleles were set to 1 (red), both MI and LD were low, but MI was always lower than LD (Figures S4AB). The effect described in Figure S3 was clear: when in the presence of dominance of one allele over the other three, LD increases with dominance (i.e. decreasing Shannon, Figure S3B). This is not observed for MI, for which increasing dominance (i.e. decreasing Shannon) leads to MI converging to zero.

For site pairs in which two out of four alleles were set to 1 (blue), LD and MI behaved the most differently. Both MI and LD are seen to peak at intermediate levels of diversity but there is a large subset of site pairs with lower diversity that presented much higher LD than MI (Figure S4ABC). Such points are the ones in which, although two alleles out of four are not set to 1 (minimum), one of them had a randomly assigned low count. That is, in effect, one of the two alleles not at 1 was dominating in frequency. In Figures S4DE we present this effect with further detail (see text below).

For site pairs in which one out of four alleles were set to 1 (green), as well as the site pairs in which allele counts were completely random (grey), LD and MI behaved very similarly (Figure S4AB). This is because in this scenario, dominance of one allele over the others is likely to be a rarer simulated event. LD was nonetheless always larger than MI (Figure S4C).

For completeness, we simulated further *in silico* allele counts in the context of the site pairs with two out of four alleles set to 1 (blue). For this, we exemplify show how LD and MI change as the particular count of a single allele changes incrementally. Figure S5A presents the site pair ***J={RY:95, SY:190, RX:712, SX:1}*** (in orange), and Figure S5B presents the site pair ***K={RY:986, SY:1, RX:1, SX:979}*** (also in orange). These site pairs are selected for being very different; in particular, ***J*** presents intermediate MI and LD, while pair ***K*** has maximum MI and LD.


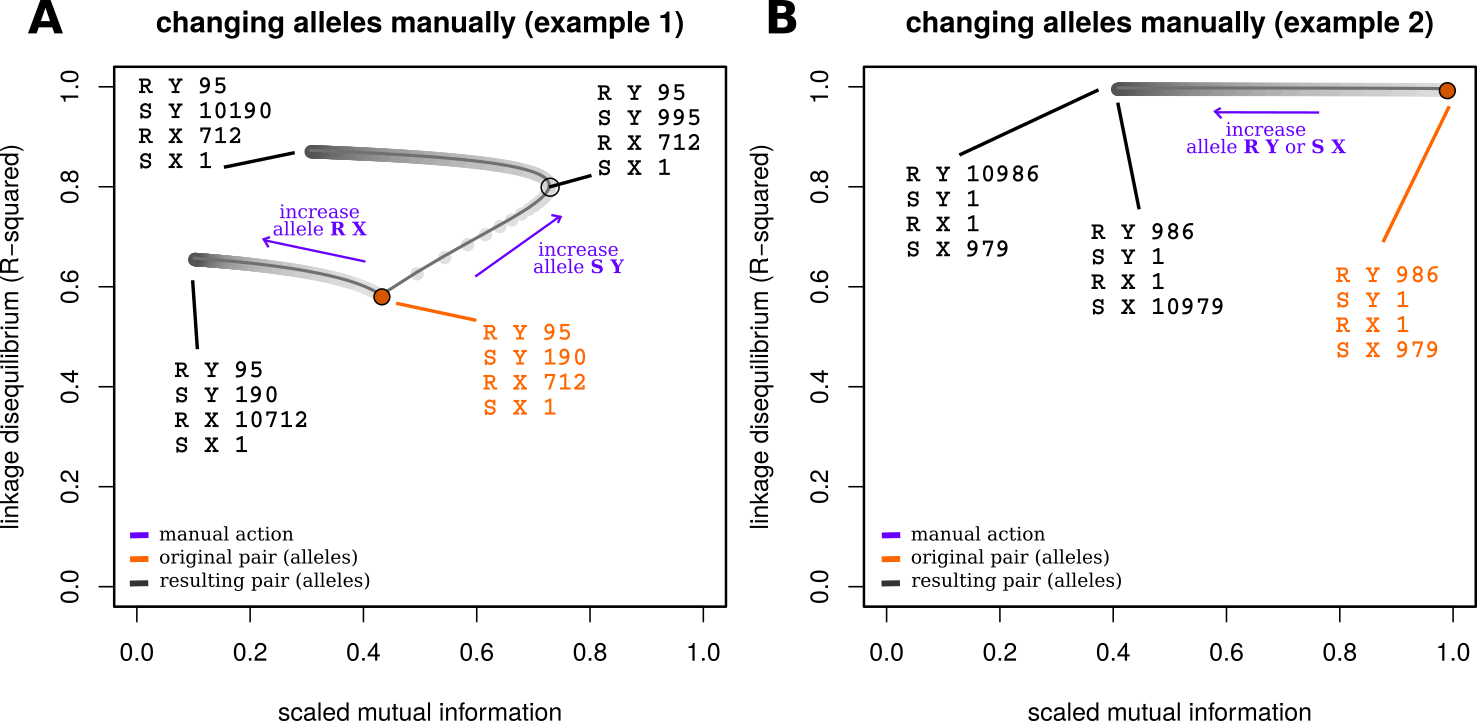


**Figure S5 - measures of linkage disequilibrium and mutual information for in silico pairs of sites with one allele’s count being incrementally changed. (A)** Site pair ***J={RY:95, SY:190, RX:712, SX:1}*** has the count of two of its alleles (S*Y, RX*) changed incrementally and independently. Mutual information (scaled, MI) and linkage disequilibrium (R^2^, LD) are calculated for each allele combination. The purple text and arrows mark the incremental changes acted. The grey points mark the incremental changes of the original orange pair in terms of MI and LD. **(B)** Site pair ***K={RY:986, SY:1, RX:1, SX:979}*** has the count of two of its alleles (*RY, SX*) changed incrementally and independently. Mutual information (scaled, MI) and linkage disequilibrium (R^2^, LD) are calculated for each allele combination. The purple text and arrows mark the incremental changes acted. The grey points mark the incremental changes of the original orange pair in terms of MI and LD.

When we incrementally changed allele *SY:190* up to *SY:10190* in **site pair *J*** (Figure S5A), both MI and LD initially increased up to the point in which *SY* and *RX*, two non-overlapping alleles were dominating (*J={RY:95, SY:995, RX:712, SX:1}*). After this point, further increments in *SY* count continued to increase LD but significantly decreased MI (to a lower level than presented by ***J*** originally). When we changed allele *RX* instead (Figure S5A), increasing the allele’s already dominant frequency, a similar effect was seen, as LD increased but MI decreased (to a lower level than presented by ***J*** originally). These exercises demonstrate how dominance of one allele may lead to high LD but low MI. If the results in the main text used LD instead of MI, high linkage as a proxy for immune selection would be likely to select site pairs such as *J={RY:95, SY:10190, RX:712, SX:1}*, which does not maximize the *strain theory’s* predictions of non-overlapping combinations of alleles as structured by strong immune selections. Again, dominance of one allele and therefore linkage disequilibrium is not a signature of non-overlap.

When we changed alleles *RY:986* up to *RY:10986*, or *SX:986* up to *SX:10986*, in **site pair *K*** (Figure S5B), essentially making one allele dominant when previously was not, MI decreased significantly while LD remained near maximum. This example further illustrates that part of what makes a site pair high on MI is the presence of non-overlapping combinations of alleles – the essential signature we set to search for, based on the theoretical predictions of *strain theory*. A site pair with alleles *{RY:10986, SY:1, RX:1, SX:979}* still presents non-overlap and therefore scores at intermediate MI, but should not be preferred to site pair ***K={RY:986, SY:1, RX:1, SX:979}*** which appears perfectly balanced and non-overlapping.

# Other supplementary figures

| 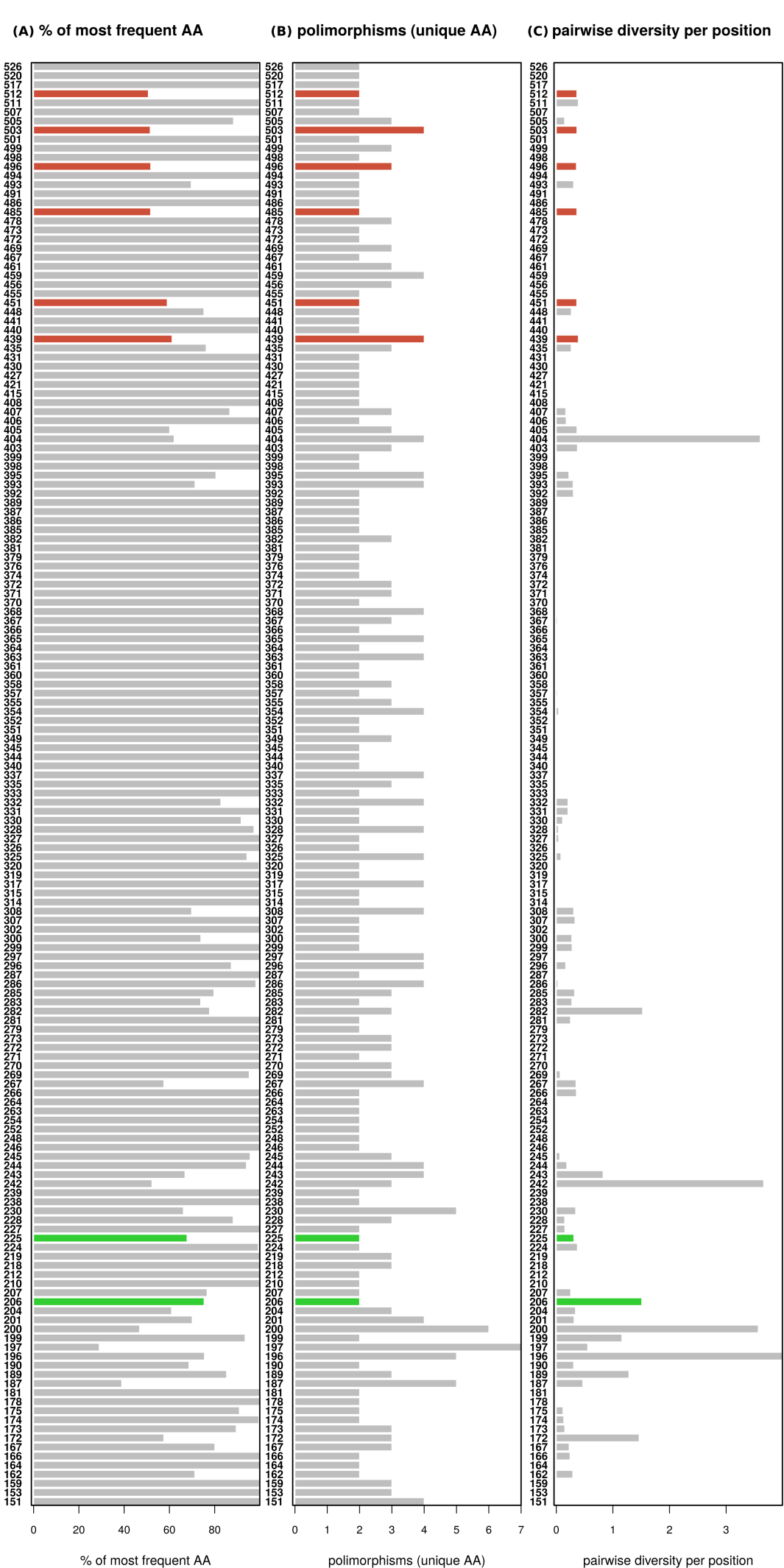 | **Figure S6 - polymorphism and diversity per site.**  For all subplots, only non-conserved sites are shown (i.e. sites that are at least dimorphic). The values here presented are supplied in supplementary Table S3, calculated before the cutoff of 2.5% frequency was applied (used for the results of the main text). Sites identified as relevant in the main text are colored in red and green. The choice of color is related to Figure 1 of the main text.  **(A)** For each site, the most common amino acid (AA) is identified, and its frequency is shown across all sequences. Note here that all displayed sites are at least dimorphic, such that sites appearing to present 100% will actually not achieve that level (see Table S3 for values).  **(B)** Number of polymorphims found per site (number of unique AA). A concentration of diversity around domain I (DI, positions ~ 150-320) is seen, as described in other studies [27]⁠.  **(C)** Pairwise genetic diversity per site, calculated in R using the function dist.ml from the package phangorn [28]⁠, and evolution model F81. |
| --- | --- |

| **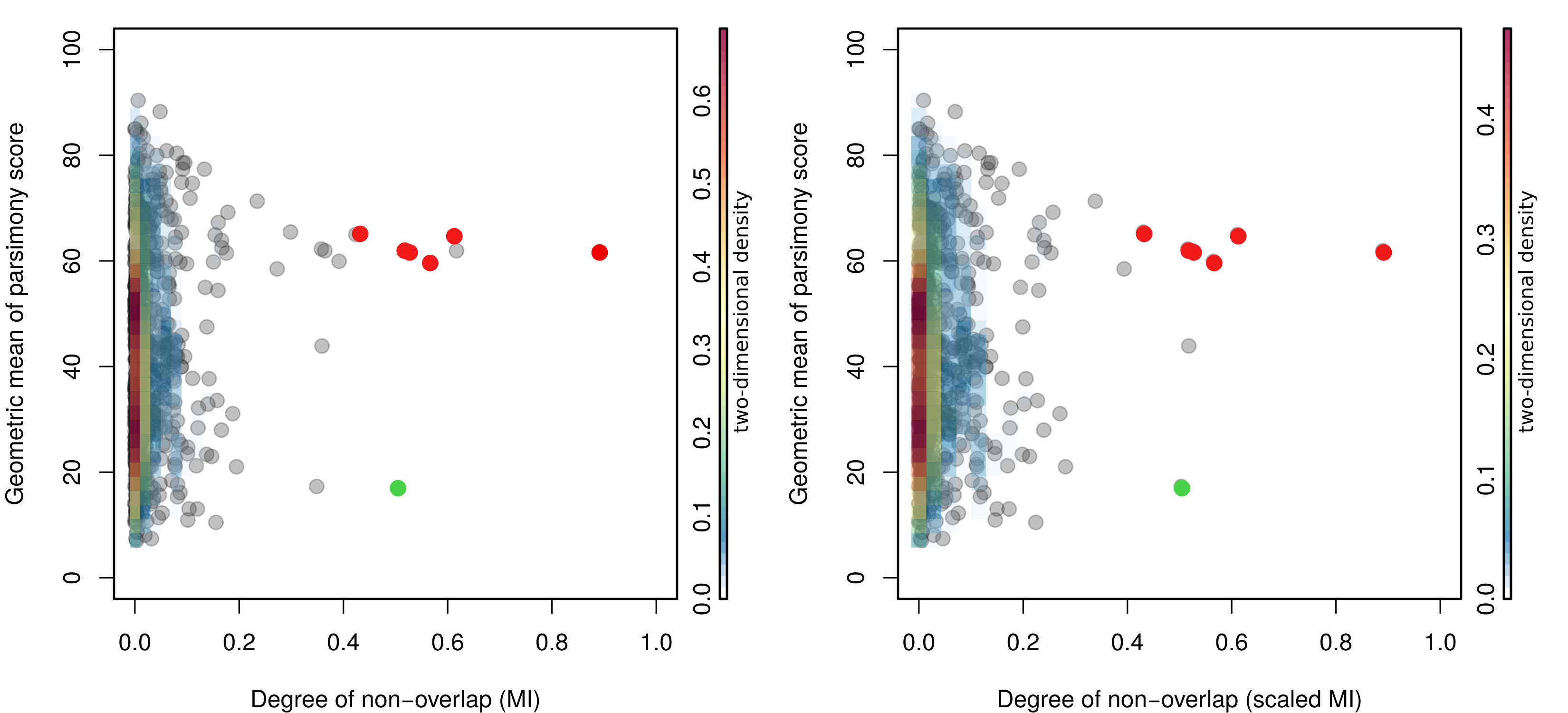** |
| --- |
| **Figure S7 – Empirical geometric mean of parsimony scores (GMPS).** GMPS is plotted against MI (left) and scaled MI (right) for all of the dimorphic pairs of sites described in the main text (see Figure 1), and Figures S1, S2 and S3 (colored the same way in green and red). These values are included in supplementary Table S2. The color key (scale) in each panel is the two-dimensional density of the points, with red for maximum observed and light blue for minimum. |

| 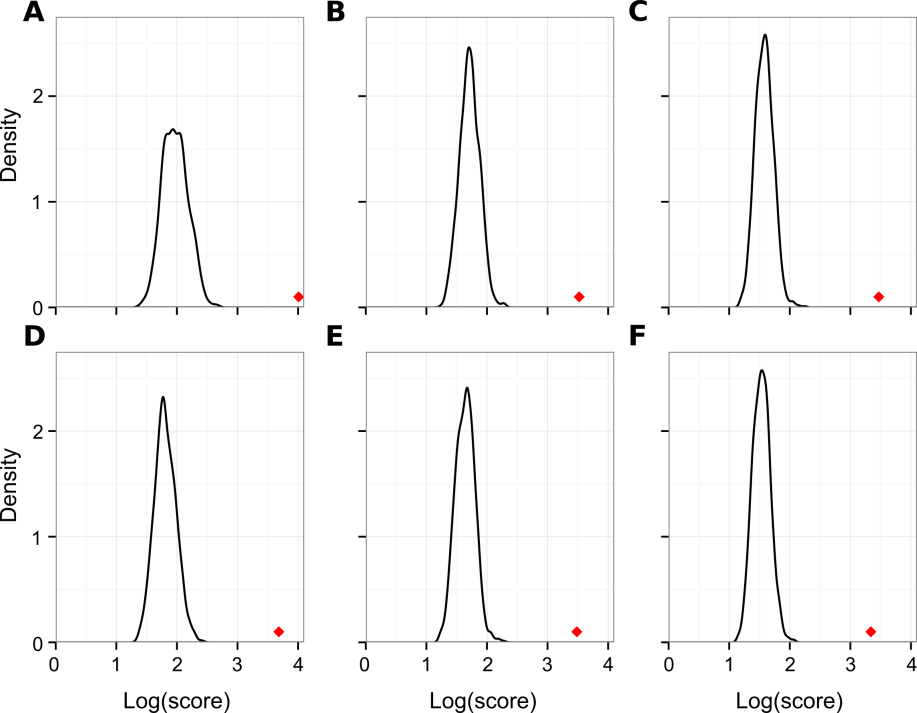 | **Figure S8 - distributions of S scores.**  Distributions are shown for the pairs of sites ranking 1 , 2 , 3 , 4 , 5 and 6 (A, B, C, D, E, F, respectively) among the 1000 simulated sets of sequences (this ranking relates to the pairs on Table 1, ordered by highest MI scores, with subplot A the highest, in the main text). For each, the equivalently ranked pair among the empirical data is shown by the red symbol (scores on Table S2).  Following suitable Box‐Cox transformations of these distributions, the red dots are 7.00, 7.44, 8.79, 8.54, 7.66 and 8.56 standard deviations away from the means of the respective simulated distribution. We are thereby able to reject the hypothesis that these scores may arise by neutral population genetic processes. For any site‐pair, a more stringent test would be to compare with the distribution of top ranked site‐pairs from the simulations: this is effectively what is shown in Figure 2 of the main text. |
| --- | --- |

| 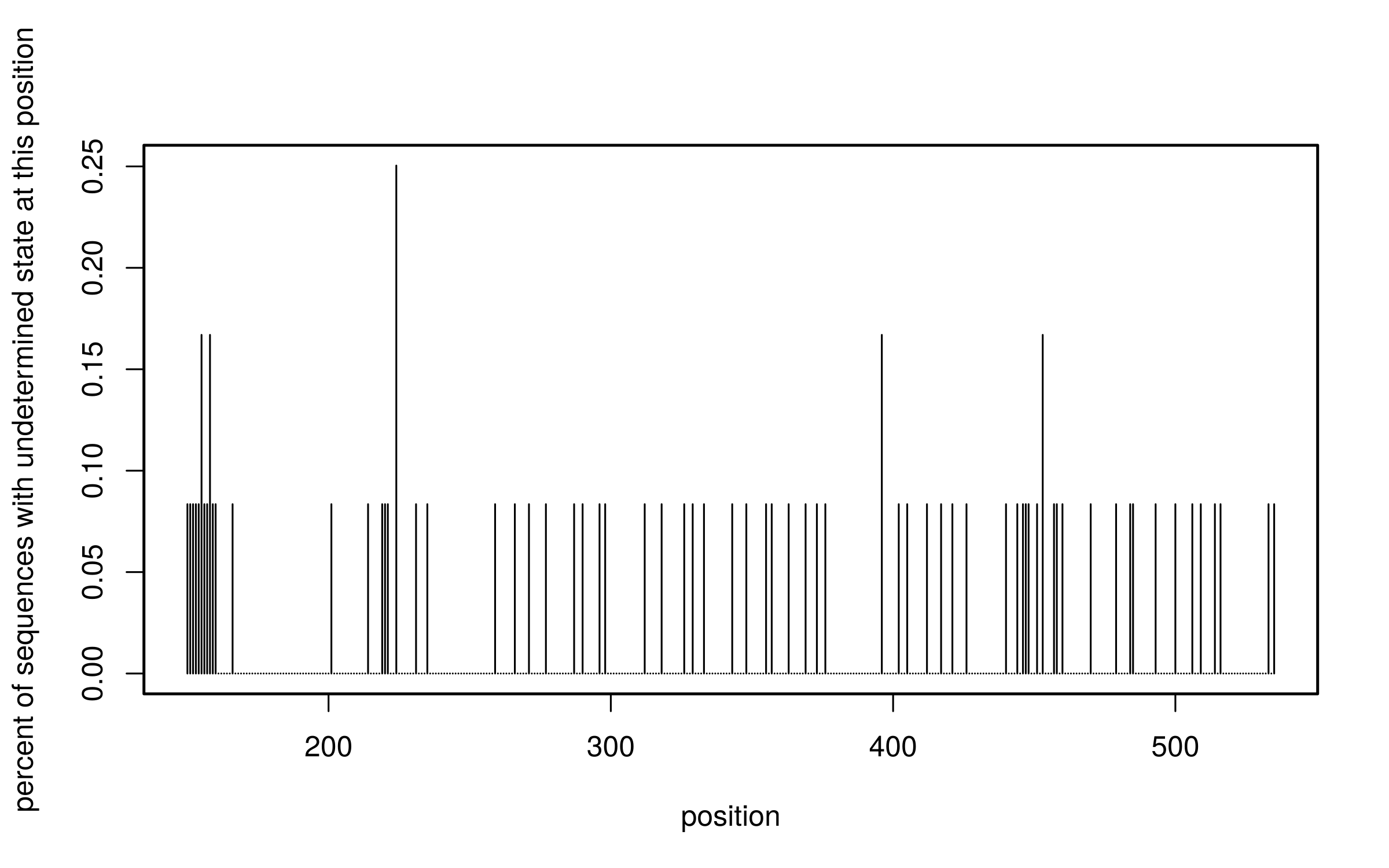 | **Figure S9 – Percent of sequences with undetermined states at each site of the aligment.**  For each position (site) in the alignment, the percentage of undetermined amino acids is calculated. An undetermined amino acid is considered to be the characters ‘-’ (gap) or ‘X’ (unknown). |
| --- | --- |

# References

[1] S. Gupta, M. C. Maiden, I. M. Feavers, S. Nee, R. M. May, and R. M. Anderson, “The maintenance of strain structure in populations of recombining infectious agents.,” *Nat. Med.*, vol. 2, no. 4, pp. 437–42, Apr. 1996.

[2] S. Gupta, N. M. Ferguson, and R. M. Anderson, “Chaos, Persistence, and Evolution of Strain Structure in Antigenically Diverse Infectious Agents,” *Science (80-. ).*, vol. 280, no. 5365, pp. 912–915, May 1998.

[3] J. R. Gog and B. T. Grenfell, “Dynamics and selection of many-strain pathogens.,” *Proc. Natl. Acad. Sci. U. S. A.*, vol. 99, no. 26, pp. 17209–17214, Dec. 2002.

[4] M. G. M. Gomes, G. F. Medley, and D. J. Nokes, “On the determinants of population structure in antigenically diverse pathogens.,” *Proc. Biol. Sci.*, vol. 269, no. 1488, pp. 227–33, Feb. 2002.

[5] S. Kryazhimskiy, U. Dieckmann, S. a. Levin, and J. Dushoff, “On state-space reduction in multi-strain pathogen models, with an application to antigenic drift in influenza A,” *PLoS Comput. Biol.*, vol. 3, no. 8, pp. 1513–1525, Aug. 2007.

[6] J. Lourenço and M. Recker, “Dengue serotype immune-interactions and their consequences for vaccine impact predictions.,” *Epidemics*, vol. 16, pp. 40–8, Sep. 2016.

[7] J. Lourenço and M. Recker, “Natural, Persistent Oscillations in a Spatial Multi-Strain Disease System with Application to Dengue,” *PLoS Comput. Biol.*, vol. 9, no. 10, p. e1003308, Oct. 2013.

[8] J. Lourenço, P. S. P. S. Wikramaratna, and S. Gupta, “MANTIS: an R package that simulates multilocus models of pathogen evolution.,” *BMC Bioinformatics*, vol. 16, no. 1, p. 176, 2015.

[9] M. Recker, O. G. Pybus, S. Nee, and S. Gupta, “The generation of influenza outbreaks by a network of host immune responses against a limited set of antigenic types.,” *Proc. Natl. Acad. Sci. U. S. A.*, vol. 104, no. 18, pp. 7711–7716, May 2007.

[10] P. S. Wikramaratna, O. G. Pybus, and S. Gupta, “Contact between bird species of different lifespans can promote the emergence of highly pathogenic avian influenza strains.,” *Proc. Natl. Acad. Sci. U. S. A.*, vol. 111, no. 29, pp. 10767–72, Jul. 2014.

[11] C. P. Thompson *et al.*, “A potent neutralizing epitope of limited variability in the head domain of haemagglutinin as a novel influenza vaccine target,” *bioRxiv*, Jan. 2018.

[12] C. O. Buckee, M. Recker, E. R. Watkins, and S. Gupta, “Role of stochastic processes in maintaining discrete strain structure in antigenically diverse pathogen populations.,” *Proc. Natl. Acad. Sci. U. S. A.*, vol. 108, no. 37, pp. 15504–15509, Sep. 2011.

[13] P. S. Wikramaratna *et al.*, “Effects of neutralizing antibodies on escape from CD8+ T-cell responses in HIV-1 infection.,” *Philos. Trans. R. Soc. Lond. B. Biol. Sci.*, vol. 370, no. 1675, pp. 471–484, 2015.

[14] C. O. Buckee, S. Gupta, P. Kriz, M. C. J. J. Maiden, and K. A. Jolley, “Long-term evolution of antigen repertoires among carried meningococci.,” *Proc. Biol. Sci.*, vol. 277, no. 1688, pp. 1635–1641, Jun. 2010.

[15] E. R. Watkins and M. C. J. Maiden, “Metabolic shift in the emergence of hyperinvasive pandemic meningococcal lineages,” *Sci. Rep.*, vol. 7, no. 1, p. 41126, Dec. 2017.

[16] J. S. Bennett, E. A. L. Thompson, P. Kriz, K. A. Jolley, and M. C. J. Maiden, “A common gene pool for the Neisseria FetA antigen.,” *Int. J. Med. Microbiol.*, vol. 299, no. 2, pp. 133–9, Feb. 2009.

[17] J. Lourenço, W. Tennant, N. R. Faria, A. Walker, S. Gupta, and M. Recker, “Challenges in dengue research: A computational perspective,” *Evol. Appl.*, vol. 11, no. 4, pp. 516–533, Apr. 2018.

[18] E. R. Watkins *et al.*, “Vaccination Drives Changes in Metabolic and Virulence Profiles of Streptococcus pneumoniae.,” *PLoS Pathog.*, vol. 11, no. 7, p. e1005034, 2015.

[19] J. Lourenço *et al.*, “Lineage structure of Streptococcus pneumoniae may be driven by immune selection on the groEL heat-shock protein,” *Sci. Rep.*, vol. 7, no. 1, 2017.

[20] L. B. Heilprin, “Information Theory and Statistics. Solomon Kullback. Wiley, New York,” *Science (80-. ).*, vol. 131, no. 3404, pp. 917–918, Mar. 1960.

[21] G. B. Gloor, L. C. Martin, L. M. Wahl, and S. D. Dunn, “Mutual information in protein multiple sequence alignments reveals two classes of coevolving positions.,” *Biochemistry*, vol. 44, no. 19, pp. 7156–65, May 2005.

[22] D. Y. Little and L. Chen, “Identification of coevolving residues and coevolution potentials emphasizing structure, bond formation and catalytic coordination in protein evolution,” *PLoS One*, vol. 4, no. 3, 2009.

[23] L. C. Martin, G. B. Gloor, S. D. Dunn, and L. M. Wahl, “Using information theory to search for co-evolving residues in proteins.,” *Bioinformatics*, vol. 21, no. 22, pp. 4116–24, Nov. 2005.

[24] C. S. Carlson, M. A. Eberle, M. J. Rieder, Q. Yi, L. Kruglyak, and D. A. Nickerson, “Selecting a Maximally Informative Set of Single-Nucleotide Polymorphisms for Association Analyses Using Linkage Disequilibrium,” *Am. J. Hum. Genet.*, vol. 74, no. 1, pp. 106–120, 2004.

[25] J. C. Mueller, “Linkage disequilibrium for different scales and applications.,” *Brief. Bioinform.*, vol. 5, no. 4, pp. 355–364, 2004.

[26] M. Scholz and D. Hasenclever, “Comparison of Estimators for Measures of Linkage Disequilibrium,” *Int. J. Biostat.*, vol. 6, no. 1, Jan. 2010.

[27] T. Bai *et al.*, “Structure of AMA1 from Plasmodium falciparum reveals a clustering of polymorphisms that surround a conserved hydrophobic pocket,” *Proc. Natl. Acad. Sci.*, vol. 102, no. 36, pp. 12736–12741, 2005.

[28] K. P. Schliep, “phangorn: Phylogenetic analysis in R,” *Bioinformatics*, vol. 27, no. 4, pp. 592–593, 2011.
